# Comparative Analysis of Gut Microbiota in Centenarians and Young People: Impact of Eating Habits and Childhood Living Environment

**DOI:** 10.3389/fcimb.2022.851404

**Published:** 2022-03-15

**Authors:** Epp Sepp, Imbi Smidt, Tiiu Rööp, Jelena Štšepetova, Siiri Kõljalg, Marika Mikelsaar, Indrek Soidla, Mare Ainsaar, Helgi Kolk, Mirjam Vallas, Madis Jaagura, Reet Mändar

**Affiliations:** ^1^ Department of Microbiology, Institute of Biomedicine and Translational Medicine, Faculty of Medicine, Tartu University, Tartu, Estonia; ^2^ Institute of Social Studies, Faculty of Social Sciences, University of Tartu, Tartu, Estonia; ^3^ Department of Internal Medicine, Institute of Clinical Medicine, University of Tartu, Tartu, Estonia; ^4^ Department of Traumatology, Tartu University Hospital, Tartu, Estonia; ^5^ BioCC OÜ, Tartu, Estonia; ^6^ Center of Food and Fermentation Technologies, Tallinn, Estonia

**Keywords:** microbiota of centenarians, good cognitive function, childhood environment, eating habits, gut enterotype

## Abstract

The composition of centenarians’ gut microbiota has consistently been used as a model for healthy aging studies. However, there is an incomplete understanding of how childhood living conditions and eating habits affect the development and composition of gastrointestinal microbiota in centenarians with good cognitive functions. We compared the gut microbiota as well as the living and eating habits of the oldest-old group and the young people group. The richness and diversity of microbiota and the abundance of hereditary and environmental microbes were higher in people with longevity than young people. People with longevity ate more potatoes and cereal products. In their childhood, they had more exposure to farm animals and did not have sewers compared with young people. Young people’s gut microbiota contained more butyrate-producing bacteria and bacteria that characterized an animal-based Western diet. These results expand our understanding of the effects of childhood environment and diet on the development and stability of the microbiota in people with longevity.

## Introduction

The world’s population is aging and this poses a significant burden to both the economy and healthcare systems around the world. Demographic projections suggest that in the year 2050 there will be 3.7 million centenarians across the globe (United Nations and Department of Economic and Social Affairs, Population Division, 2015). The problem arises on how to sustain health in the aging process. Centenarians (around 100 years of age) pertain to people with the longest life span as a result of their conscious effort to maintain their health.

Several studies have explored the role of gut microbiota in healthy aging and longevity (O'Toole and Jeffery, 2018). The composition of the human gut microbiota is mainly linked to human genetic background and epigenetic modulators such as environment, diet, aging, and development of society (Shenderov, 2012; Mangiola et al., 2018; Nagpal et al., 2018; Coman and Vodnar, 2020).

The human gut bacterial community displays more than 2,000 different species with the abundance of some of them up to 10^10–11^ colony-forming units per gram in numbers (Almeida et al., 2019). Their diverse roles, such as participation in nutrition, maintaining immune homeostasis, colonization resistance against pathogens, regulation of the intestinal endocrine function, and permeability, are well known.

The development of human gastrointestinal microbiota is a gradual process, and by the age of 3–4 years, an individual develops a mature gastrointestinal microbiota and enterotype, which remains relatively stable until the end of life (Turnbaugh et al., 2009; Arrieta et al., 2014; Bergstrom et al., 2014). The enterotypes describe the phylogenetic and functional variation of the gut microbiota that may affect different biomarkers of the host in different ways (Arumugam et al., 2011). For example, the *Prevotella* enterotype is characterized by hydrolases effective in the degradation of plant fibers and low lipolytic and proteolytic fermentation potential and the *Bacteroides* enterotype has enzymes specialized in the degradation of animal carbohydrates and increased saccharolytic and proteolytic capacity (Vieira-Silva et al., 2016). It is possible that the host survival granting features can be predicted by the composition and the metabolites of individual gut microbiota starting from youth.

The composition of the gut microbiota of centenarians and young adults living in the same area and having similar eating habits may still be different (Biagi et al., 2016; Kong et al., 2016; Wang et al., 2019). The gut microbiota of elderly people displays greater interindividual variation than that of younger adults (Claesson et al., 2012; Nagpal et al., 2018). This large variation may be attributed to multiple underlying factors of physiological aging of the host and contributing comorbidities, mobility disorders, frailty, and polypharmacy. In addition to a variety of somatic diseases, many elderly people experience cognitive impairment. However, some centenarians maintain good cognitive health. There is a possibility that these supercontrol centenarians recognize the contribution of a healthy gut microbiota to cerebral functions (Ticinesi et al., 2018). The gut microbiota serves as an important candidate as a marker for healthy aging; therefore, its composition in centenarians has consistently been used as a model for healthy aging studies (Kong et al., 2019; Wu et al., 2019). Unfortunately, to date, there have not been enough studies of intestinal microbiota of centenarians with a well-distinguished health marker such as cognitive function.

The aim of this study was to compare the gut microbiota as well as the living and eating habits of Estonian centenarians and oldest-old people born before the year 1920 with that of young people born in the late 1990s. The centenarians and oldest-old people were selected according to preserved cognitive function.

## Material and Methods

### Study Population

This comparative study of the gut microbiota in oldest-old (including centenarians and oldest-old people) and young groups was conducted in 2018. Young people (*n* = 25) were born in the late 1990s and centenarians and oldest-old people (*n* = 25) before the year 1920. In centenarians and oldest-old subjects, the inclusion criteria were as follows: suitable age, voluntariness, suitable Mini-Mental test result while measuring cognitive ability (normal or mild), ability to answer the questionnaire, and no antibiotic administration during 30 days prior to the study. The exclusion criteria for young people were gastrointestinal disease, food allergy, diabetes, acute infection, use of any antimicrobial agent within the preceding 3 months, use of non-steroidal anti-inflammatory drugs, pregnancy or breastfeeding, any serious organ or systemic disease, high blood pressure, eating disorder, and weight loss of >5 kg in the prior 3 months.

Study subjects were asked about their living environment during the first 5 years of life and during the study (family size, exposure to animals, existence of water closets and sewers, eating habits). The participants were weighed and their height was measured for body mass index (BMI) calculation. Health data were obtained from a national database (Electronic Health Record) which includes all outpatient and hospital health records starting from 2010. The Ethics Committee of the University of Tartu approved the study (protocol 275/T-13).

### Sample Collection

The fecal samples were collected from all subjects by using the FecesCatcher system and stored in domestic refrigerators at −20°C before transportation to the laboratory. In the laboratory, the samples were frozen at −80°C until analyses to determine the gut microbiota.

### DNA Extraction, 16S rRNA Gene Sequencing Strategy, and Analysis

Bacterial DNA from feces was extracted with the QIAamp Power Fecal DNA kit (Qiagen, Hilden, Germany), using an ELMI Sky Line instrument (ELMI Ltd., Sweden) according to the manufacturer’s instructions.

Amplicon sequencing was conducted at the Institute of Genomics Core Facility, University of Tartu. A 460-bp region encompassing the V3 and V4 hypervariable regions of the 16S rRNA gene was amplified and sequenced using the Illumina MiSeq Reagent kit v3 in paired end 2 × 300 bp mode on an Illumina MiSeq instrument according to Klindworth et al. (2013). The library pool was quantified using an Illumina-specific KAPA Library Quant kit (Kapa Biosystems, Wilmington, MA, USA).

DNA sequence data were analyzed using BION-meta, currently an unpublished open source program, according to the authors’ instructions. A detailed description of the methods used is presented by McDonald et al. (2016). First, sequences were cleaned at both ends using a 99.5% minimum quality threshold for at least 18 of 20 bases for the 5′ end and 28 of 30 bases for the 3′ end, and then joined, followed by removal of contigs shorter than 350 bp. The sequences were cleaned from chimeras and clustered by 95% oligonucleotide similarity (k-mer length of 8 bp, step size 2 bp). Lastly, consensus reads were aligned to the SILVA reference 16S rDNA database (v123) using a word length of 8 and a similarity cutoff of 90.

### Statistical Analysis

Statistical analyses were performed using R version 4.0.2 (The R Foundation for Statistical Computing, Vienna, Austria) statistical software package. The data were analyzed using the Wilcoxon rank sum test, Fisher’s exact test, and Spearman and Kendall rank correlation. Eating habits were compared by Fisher’s exact test and correlated with microbiota composition by Kendall rank correlation coefficient. Statistical significance was declared as *P <*0.05. The diversity of microbiota was described by the Shannon diversity index. The composition of the microbiota was compared by the Wilcoxon rank sum test. Associations between bacterial abundance were assessed by Spearman rank correlation analysis. For multiple testing correction, we applied the Benjamin–Hochberg method and considered the significance level to be 0.1.

## Results

### Characteristics of Centenarians and Oldest-Old Subjects as the “Oldest-Old Group”

Based on the Mini-Mental test, 21 oldest-old subjects had normal cognition (24–30 points) and 4 had mild (19–23 points) cognitive impairment. Most of the participants had diagnoses of physical disorders, while the only mental health diagnosis in the group of oldest-old was sleeping disorders. No diagnoses of dementia or other psychiatric disorders were detected.

A number of chronic non-communicable diseases were on average 4.7 (range 0–10) among the oldest-old subjects. The most frequent diagnoses were hypertension and chronic cardiac insufficiency diagnosed in 21 and 17 cases, respectively. Seven people were diagnosed with ischemic heart disease and five had atrial fibrillation. Sixteen people out of 21 used one antihypertensive medication and five persons used two antihypertensive medications resulting in normal or slightly low blood pressure. According to routine blood analysis, three older participants had mild anemia.

### Living Environment and Eating Habits of the Participants

There were no differences in terms of gender, family size, and body mass index between the two investigated groups (oldest-old subjects and young people) during the study (**Table 1**). During the first 5 years of life, the family size in both groups was similar. In the oldest-old group, there was more exposure to farm animals like horses, cows, and sheep, and also they did not have water closets and/or sewers, except for one subject (**Table 1**). Usage of water closets and/or sewers became possible for the oldest-old group when they were over 40 years old.

**Table 1:** Basic characteristics and living environment of the study subjects.

Groups		Oldest-old (n=25)	Young (n=25)	p- value*
Grouping characteristic				
	**Age (years)**			<0.001
	min-max	96-105	19-23	
	median	99	20	
	mean	99	21	
Sex	Female/Male	18/7	13/12	0.244
During the study				
	**Living environment**			0.005
	alone	13	8	
	with spouse	4	4	
	with family	5	4	
	with peers	0	9	
	in nursing home	3	0	
	**BMI**			0.065
	min-max	19.1-35.1	17.8-32.4	
	median	26	23.2	
	mean	25.4	23.6	
First 5 years of life (number of people)				
	**Family size**			0.604
	min-max	3-11	2-7	
	median	4	4	
	mean	4.8	4.1	
	**Exposure to animals**			<0.001
	farm	19	3	
	pet	4	14	
	no	2	8	
	**Existence of water closets**			<0.001
	yes	0	25	
	no	25	0	
	**Existence of sewers**			<0.001
	yes	1	25	
	no	24	0	

*Wilcoxon rank sum test for BMI, Fisher’s exact test for remaining characteristics.

During the study, the centenarians and oldest-old people consumed potatoes (*p* = 0.047) and cereal products (white and black bread, porridge; *p* < 0.001) more often than young people. There were no differences found in the consumption of meat and meat products, vegetables, milk, and dairy products between the investigated groups (**Supplementary Table**).

### Richness, Diversity, and Distribution of Fecal Microbiota Composition

**Figure 1:**
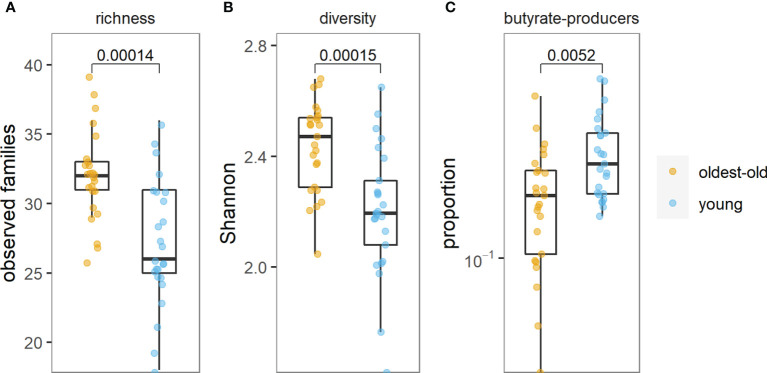
Richness of microbiota **(A)**, diversity of microbiota **(B)**, and abundance of butyrate-producing bacteria **(C)** in the oldest-old and young groups.

**Figure 2:**
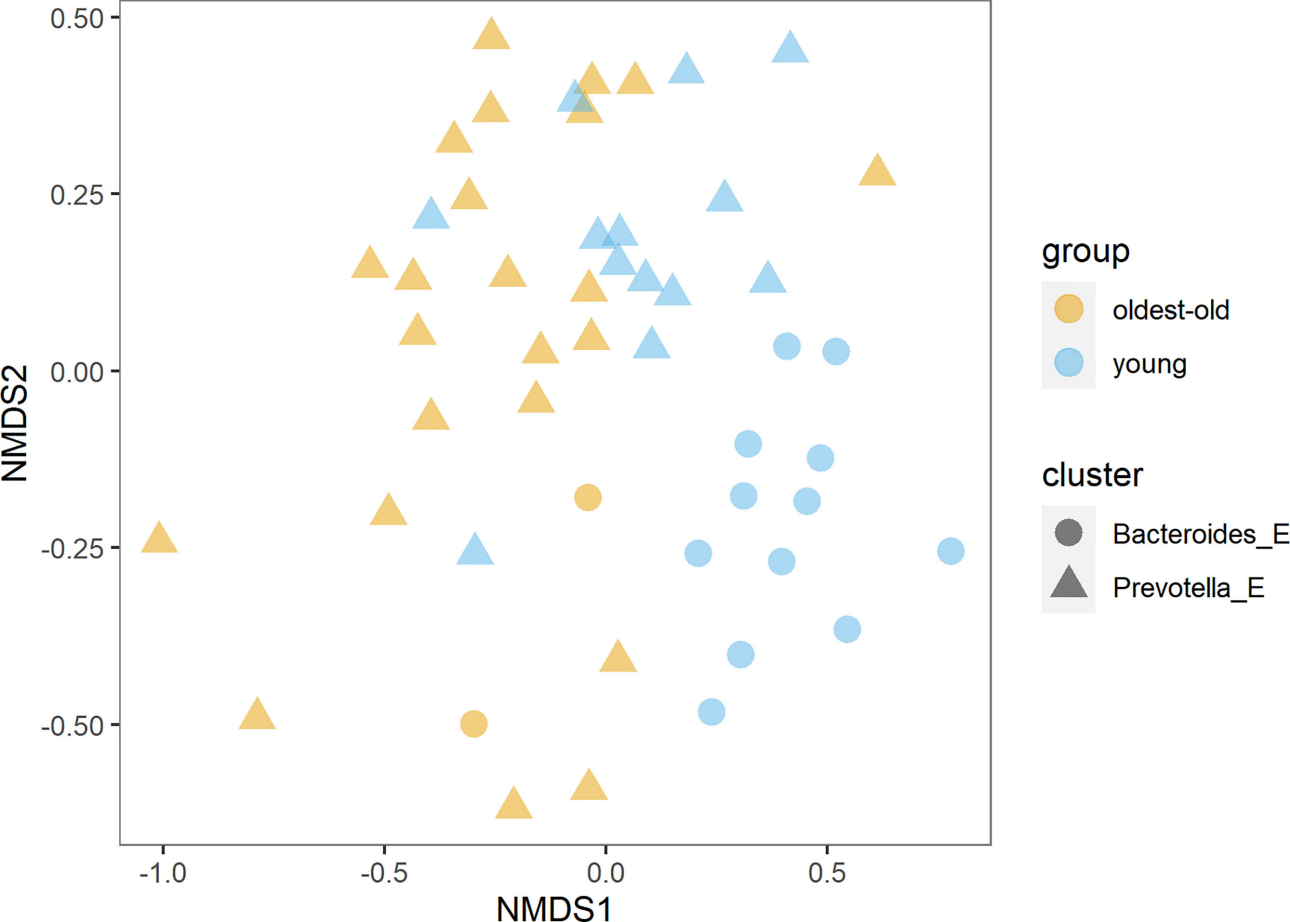
Distribution of enterotypes in the oldest-old and young groups (NMDS, non-metric multidimensional scaling).

We found more than 150 families of bacteria belonging to 27 different phyla in this study. The richness and diversity of microbiota were significantly higher in centenarians and oldest-old than in young people (**Figure 1**). The *Prevotella* enterotype was predominant in centenarians and oldest-old people, while the *Bacteroides* enterotype was predominant in young people (**Figure 2**).

The abundance of butyrate-producing bacteria was lower in centenarians and oldest-old people than in young people (*p* = 0.005) (**Figure 1** and **Supplementary Figure**).

### Fecal Sample Taxa Composition

**Figure 3:**
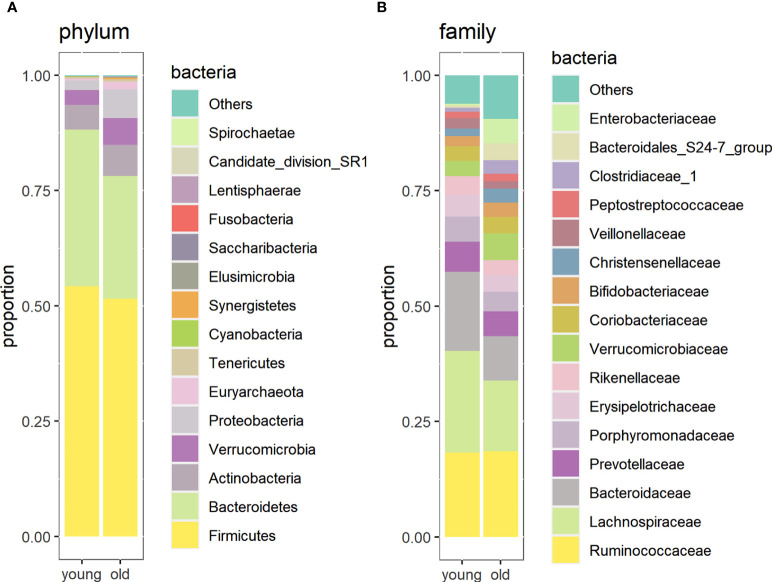
Relative abundance of gut microorganisms in the oldest-old and young groups at the phylum level **(A)** and family level **(B)**.

At the phylum level, *Firmicutes* was the most abundant in both groups, followed by *Bacteroidetes* and *Actinobacteria*. The relative abundance of *Euryarchaeota* (*p* = 0.0039), *Synergistetes* (*p* = 0.0038), and *Proteobacteria* (*p* = 0.052) was higher in centenarians and oldest-old people, whereas that of *Bacteroidetes* (*p* = 0.038) was higher in young people (**Figure 3A**).

At the family level, the most abundant were *Ruminococcaceae*, *Lachnospiraceae*, *Bacteroidaceae*, and *Prevotellaceae*. The abundance of *Christensenellaceae* (p = 0.02), *Clostridiaceae* (p = 0.003), *Enterobacteriaceae* (p = 0.0007), *Methanobacteriaceae* (p = 0.003), and *Synergistaceae* (p = 0.024) was higher in the longevity group than in the young group. At the same time, the abundance of Bacteroidaceae (p = 0.036), *Alcaligenaceae* (p = 0.013), *Erysipelotrichaceae* (p = 0.018), and *Lachinospiraceae* (p = 0.01) was higher in young people than in centenarians and oldest-old people (**Figure 3B**). In the oldest-old group, the consumption of milk and/or dairy products was positively correlated with the abundance of Prevotellaceae (r = 0.440; p = 0.028) and the consumption of cereal products with the abundance of *Enterobacteriaceae* (r = 0.415; p = 0.039). In young people, the consumption of vegetables and salads was positively correlated with the abundance of *Flavobacteriaceae* (r = 0.436; p = 0.029) and the consumption of acidified food with Leuconostocaceae (r = 0.480; p = 0.015). However, no statistically significant correlations between microbiota composition and eating habits were retained after applying correction for multiple testing.

**Figure 4:**
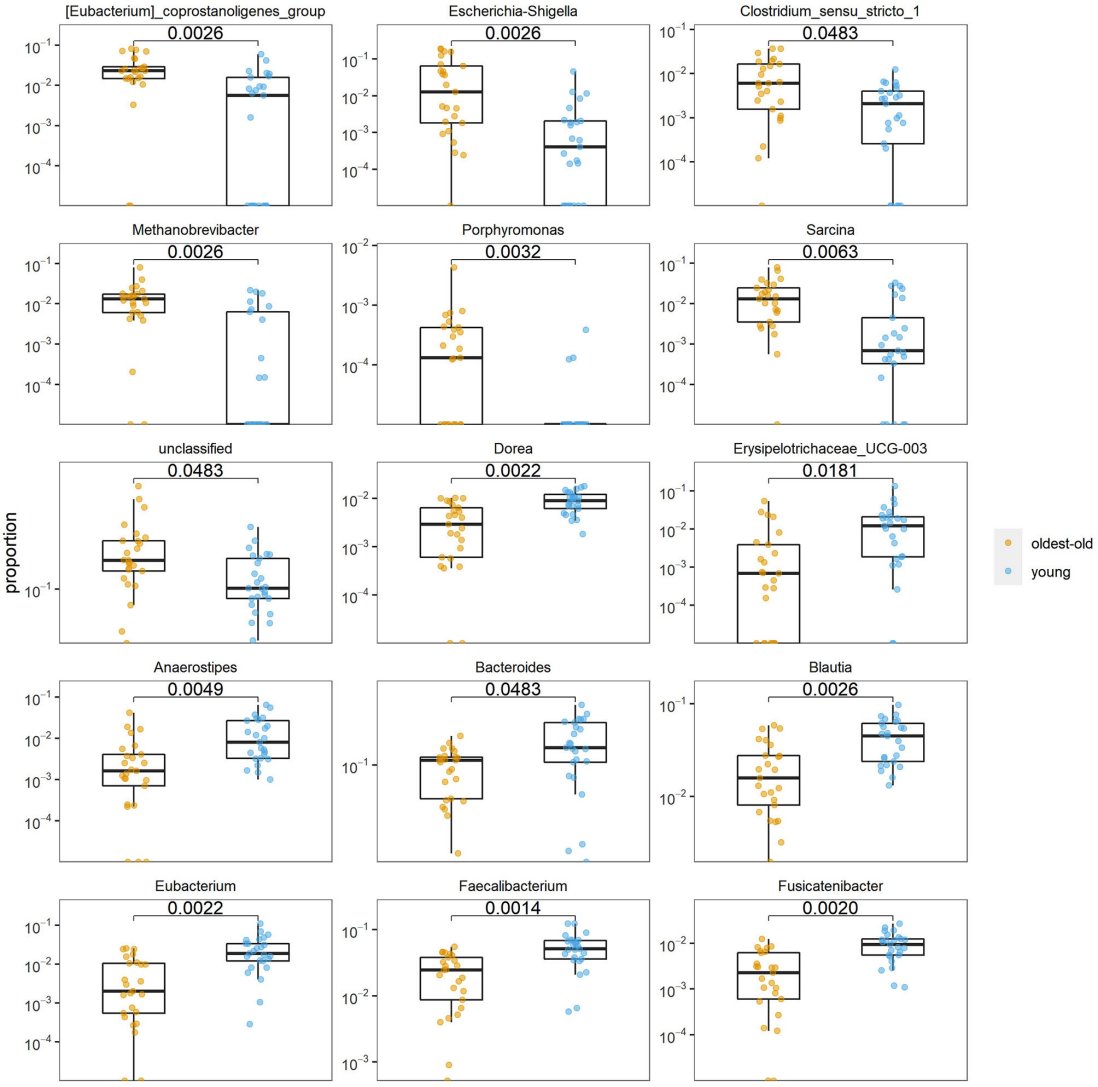
Differences at the genus level of the intestinal microbiota in the oldest-old and young groups.

At the genus level, the abundance of *Eubacterium coprostanoligenes* group (*p* = 0.003), *Escherichia–Shigella* group (*p* = 0.003), *Clostridium sensu stricto* (*p* = 0.048), *Methanobrevibacter* (*p* = 0.003), *Porphyromonas* (*p* = 0.003), and *Sarcina* (*p* = 0.006) was higher in the longevity group than in the young group, but that of *Anaerostipes* (*p* = 0.005), *Bacteroides* (*p* = 0.048), *Blautia* (0.003), *Dorea* (*p* = 0.002), *Eubacterium* (*p* = 0.002), *Faecalibacterium* (*p* = 0.001), and *Fusicatenibacter* (*p* = 0.002) was lower in the longevity group than in the young group (**Figure 4**).

**Figure 5:**
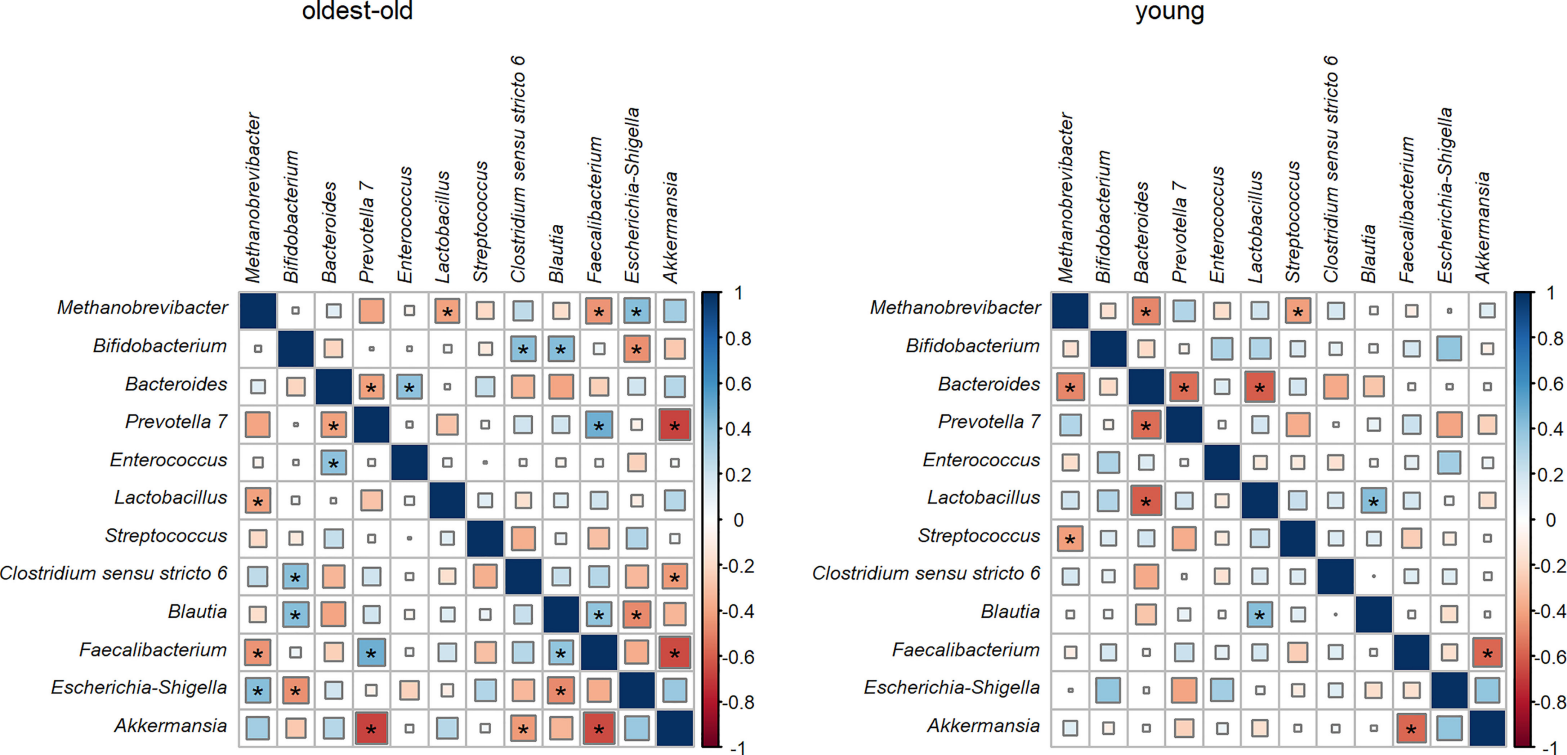
The correlation between different bacterial abundance in the oldest-old and young groups (associations which were statistically significant without adjustment for multiple comparisons are designated by an asterisk).

In both investigated groups, a negative correlation was found between the abundance of *Prevotella* and *Bacteroides*, as well as the abundance of *Akkermansia* and *Faecalibacterium*. *Methanobrevibacter* had a negative correlation with lactic acid bacteria such as lactobacilli in the oldest-old group and with streptococcus in the young group. In young people, the relative abundance of *Bacteroides* was in negative correlation with *Methanobrevibacter* and lactobacilli. In centenarians and oldest-old people, the abundance of *Prevotella* was in negative correlation with *Akkermansia* and in positive correlation with *Faecalibacterium*. Also, in the oldest-old group, the relative abundance of *Escherichia–Shigella* had a positive correlation with *Methanobrevibacter* and a negative correlation with bifidobacteria and *Blautia.* The relative abundance of *Clostridium sensu stricto* had a positive correlation with bifidobacteria and a negative correlation with *Akkermansia* in centenarians and oldest-old people (**Figure 5**). After correction for multiple testing, statistical significance could be established only for the negative correlations of *Akkermansia* with *Faecalibacterium* and *Prevotella* in the oldest-old group.

At the species level, the centenarians and the oldest-old people had an increased relative abundance of *Bifidobacterium dentium* (*p* = 0.012), *Bifidobacterium longum* (*p* = 0.041), *Escherichia coli* (*p* = 0.025), and *Methanobrevibacter smithii* (*p* = 0.004) as compared with young people. In young people, we found a higher relative abundance of *Blautia faecis* (*p* = 0.019), *Blauta wexlerae* (*p* = 0.003), *Dorea formicigenerans* (*p* = 0.001), *Dorea longicatena* (*p* = 0.03), *Fusicatenibacter saccharivorans* (*p* = 0.001), and *Lachnoclostridium lactaris* (*p* = 0.007) from the *Lachnospiraceae* family; *Eubacterium ramulus* (*p* = 0.003) and *Eubacterium rectale* (*p* = 0.003) from the *Eubacteriaceae* family; and *Faecalibacterium prausnitzii* (*p* = 0.003) from the *Ruminococcaceae* family.

## Discussion

Our study revealed substantial differences in the gut microbiota between the Estonian longevity group and young people. The richness and diversity of microbiota were significantly higher in centenarians and oldest-old people than in young people. The *Prevotella* enterotype predominated in centenarians and oldest-old people, while the *Bacteroides* enterotype predominated in young people. This is the first study to compare childhood living conditions and eating habits affecting significantly the development and composition of gastrointestinal microbiota.

Previous studies on the gut microbiota of centenarians have revealed that it has diverse features that may be a consequence of various adaptations to aging in different geographical locations where people are exposed to different dietary, physiological, and environmental conditions (Biagi et al., 2010; Kong et al., 2016; Kim et al., 2019; Tuikhar et al., 2019; Wang et al., 2019; Wu et al., 2019). In this study, we compared the gut microbiota composition of Estonian centenarians and oldest-old people, selected according to preserved cognitive function, and of young people. Gut microbiota composition was significantly different in these groups. The richness and diversity were higher in centenarians and oldest-old people than in young people. Similar results were obtained in previous studies where Italian centenarians had higher diversity and Indian, Japanese, and Chinese centenarians had higher richness of gut microbiota than young people in these countries (Kong et al., 2016; Santoro et al., 2018).

The richness and diversity of gut microbiota are acknowledged health biomarkers, including the frailty of elderly people and some personality traits (Lei et al., 2019; Johnson, 2020). Frailer individuals have decreased richness and diversity of gut microbiota (Jackson et al., 2016; Maffei et al., 2017). The diversity is significantly and inversely correlated with biological age but not with chronological age (Maffei et al., 2017). The diversity is higher among successfully aging people (Badal et al., 2020). Also, people with larger social networks tend to have a more diverse microbiota, suggesting that social interactions may shape the microbial community of the human gut (Johnson, 2020). In our study group, half of the centenarians and oldest-old people lived alone, some with their spouse or family, and three in a nursing home. Most of the centenarians and oldest-old people had normal cognitive ability with an average MMSE (Mini-Mental State Examination) score of 26.3 out of 30. This shows that centenarians and oldest-old people are doing very well on their own, which means that they are “actively aging”. We chose the subjects in our study who were able to answer the questionnaire.

In centenarians and oldest-old people, the *Prevotella* enterotype was dominant, and in young people, the *Bacteroides* enterotype was dominant. In both groups, the abundance of *Prevotella* and *Bacteroides* was negatively correlated, and in the oldest-old group, the abundance of *Prevotella* was in positive correlation with consumption of dairy products. Enterotypes are essential biomarkers that are used to describe human “health” and behavior (Heiman and Greenway, 2016; Johnson, 2020). It has been shown that a fiber-rich diet correlates with the prevalence of *Prevotella*, *Bifidobacteria*, and *Lactobacilli*; a high-carbohydrate diet correlates with the prevalence of *Prevotella*, *Methanobrevibacter*, and *Candida*; and a high-fat and amino-acid-rich diet correlates with the prevalence of *Bacteroides* (Selber-Hnatiw et al., 2020). In our study, in centenarians and oldest-old people, *Prevotella* was in positive correlation with *Faecalibacterium.* In young people, *Bacteroides* was in negative correlation with *Methanobrevibacter* and lactobacilli. The *Bacteroides* enterotype is characterized by low richness and fewer bacterial species with overlapping functionality, suggesting that this enterotype may be more vulnerable to ecosystem disturbance (Vieira-Silva et al., 2016). The *Bacteroides* enterotype together with decreased gut microbiota richness and diversity is related with our modern Western lifestyle (Lin et al., 2013; Mosca et al., 2016). In the past decades, the prevalence of numerous intestinal and extraintestinal diseases such as overweight, diabetes mellitus, or metabolic disorders has sharply increased. This phenomenon was initially observed in Western countries and more recently in developing countries, too, where the conditions of childhood environment and diet have changed abruptly (Mosca et al., 2016).

Dietary diversity has been lost during the past 50 years, and dietary choices that exclude food products from animals or plants will narrow the microbiome further (Heiman and Greenway, 2016). The eating habits of centenarians and oldest-old people differed from those of young people. The elderly ate more potatoes and cereal products, which contain large amounts of starch, fibers, and polyphenols. These substances have a beneficial effect on the gut microbiota and their metabolites (Bibi et al., 2019). Eating potatoes and cereal increases the relative abundance of lactic acid bacteria such as bifidobacteria (Bibi et al., 2019; Baxter et al., 2019) and butyric acid-producing bacteria (Gong et al., 2018; Baxter et al., 2019; Sanchez Tapia et al., 2019). Centenarians have functional differences distinguishing their microbiota from that of young-old adults, such as increased short-chain fatty acid production (Kim et al., 2019; Wu et al., 2019). This may be due to the higher species richness of the microbiota in centenarians, which promotes cross-feeding of intestinal bacteria. In our centenarians and oldest-old people, the abundance of lactic acid bacteria such as *B. longum* and *B. dentium* was higher, but the abundance of butyric acid-producing bacteria was lower than in young people. However, they had more heritable butyric acid-producing *Cristensenellaceae* as compared with young people. Similar to our study, the relative abundance of *Cristensenellaceae* was greater in centenarians than in young people also in Italy, China, and Korea (Biagi et al., 2016; Kong et al., 2016; Kim et al., 2019). It is noteworthy that *Christensenellaceae* has been associated with human longevity. Studies have found a positive association of *Christensenellaceae* with age; therefore, it is possible that individuals born earlier may have harbored greater levels of *Christensenellaceae* compared with those born later (Goodrich et al., 2014; Waters and Ley, 2019). *Christensenellaceae* is related to normal body mass index and low cardiometabolic disease risk (Goodrich et al., 2014; Waters and Ley, 2019). In addition, the high abundance of *E. coprostanoligenes* and *B. longum* may reduce cardiometabolic risk in our oldest-old group*. Eubacterium coprostanoligenes* converts cholesterol to coprostanol, a non-absorbable sterol that is excreted by the feces (Gerard, 2013). *Bifidobacterium longum* is negatively associated with insulin resistance (Brahe et al., 2015). The more diverse the diet, the more diverse and species rich the gut microbiota and the more adaptable an individual will be to perturbations, as shown also in our previous studies (Mikelsaar et al., 2016). Maintaining a balanced diet in older age may be a key factor in promoting longevity (including reducing the risk of cardiometabolic diseases) (Badal et al., 2020).

At the same time, in young people, the abundance of *Bacteroides* and *Erysipelotrichaceae* was higher, which is characteristic of an animal-based diet consisting of high fats and proteins (Kaakoush, 2015; Rinninella et al., 2019). *Bacteroides* are non-hereditary bacteria that are most affected by the environment, including diet (Goodrich et al., 2014; Stewart et al., 2018; Coman and Vodnar, 2020). A high-calorie diet may be a causal factor for obesity and may induce changes in the functions of the gut microbiota (Aoun et al., 2020). The abundance of *Erysipelotrichaceae* is higher in obese people (Kaakoush, 2015). In young people, we saw a rise in the abundance of the *Blautia* genus, which is inversely associated with visceral fat accumulation, and *Eubacterium ramulus*, which is associated positively with insulin resistance (Brahe et al., 2015; Ozato et al., 2019). The gut microbiota is related to obesity, visceral fat, and insulin resistance that in turn are reported to be strongly associated with cardiovascular diseases and overall mortality. Also, the diversity of the gut microbiota is significantly lower in obese than in non-obese subjects (Kasai et al., 2015; Lv et al., 2019). In young people, we found lower gut microbiota diversity but higher abundance of microbes that are associated with the Western diet and overweight when compared with centenarians and oldest-old people. Nevertheless, in young people, the butyrate-producing bacteria *F. prausnitzii* and Fam. *Lachnospiraceae* showed higher abundance than in centenarians and oldest-old people. These bacteria are associated with gut barrier function (tight junction) and anti-inflammatory capability (Riedel et al., 2006; Louis and Flint, 2017). The higher abundance of butyrate-producing bacteria in young people was seen also in Chinese, Korean, and Italian centenarian’s studies (Biagi et al., 2010; Biagi et al., 2016; Kim et al., 2019; Wang et al., 2019; Wu et al., 2019). Butyrate reduces appetite, decreases insulin resistance, and is protective against obesity caused by a high-calorie diet (Li et al., 2018; Aoun et al., 2020). Body mass index did not differ between centenarians and young people. The higher abundance of butyrate-producing bacteria in young people may protect them from becoming overweight.

The centenarians and oldest-old people tended to have a higher abundance of bacteria that are able to live in an outdoor environment (soil, plants, water, animals) such as *M. smithii*, *Escherichia–Shigella* group, *Synergistaceae* family, and spore-forming bacteria like *Clostridium sensu stricto*. Similar results have been found in studies conducted in China, Italy, India, and Korea: *Methanobrevibacter* was more abundant in the centenarians from Italy, *Synergistes* for centenarians from China, *Clostridium sensu stricto* for centenarians from Italy and China, and *Escherichia–Shigella* group for centenarians from India, China, and Korea when compared with young people (Drago et al., 2012; Kim et al., 2019; Tuikhar et al., 2019; Wang et al., 2019; Wu et al., 2019). The spore-forming gut bacteria like *Clostridium sensu stricto* regulate serotonin synthesis in the gut (Yano et al., 2015). Serotonin is a neurotransmitter that modulates mood, cognition, learning, memory, and other physiological processes, and it has an important role in the etiology and treatment of depression (Young, 2007). In a nematode model, *Bacillus licheniformis* enhanced longevity. This species regulates the genes associated with serotonin signaling in nematodes and, thus, may modify aging (Park et al., 2015). A positive mood may be an important predictor of health and longevity. *Methanobrevibacter smithii* is an archaea that reduces hydrogen levels *via* the production of methane, thereby stimulating food fermentation by saccharolytic bacteria. This archaea is absent in infants and its colonization may result from environmental exposure, as well as from exposure to organic milk products and organic food during childhood (van de Pol et al., 2017). At the same time, its abundance had a negative correlation with lactic acid bacteria in both groups and a positive correlation with *Escherichia–Shigella* group in centenarians and oldest-old people. The latter colonizes the gut microbiota of humans and animals, but it is also found in soil and water (Ercumen et al., 2017; Capone et al., 2019). Its proportion is high in newborns and increases again with age (Odamaki et al., 2016). In children of developing countries, who are living in farms and have contact with animals, the counts of *Escherichia–Shigella* group (including *E. coli*) are higher than in children from developed countries (Kuang et al., 2016; Vatanen et al., 2016; Milani et al., 2017). Similar changes have also been described in the adult population (Jha et al., 2018). This group of bacteria is associated with poor sanitation and hygiene, drinking water quality, and presence or absence of water toilet and sewage (Jha et al., 2018; Capone et al., 2019; Tang et al., 2019). Our centenarians and oldest-old people were born and lived in the countryside alongside farm and domestic animals and without sewage. The environmental microbes could have colonized centenarians and oldest-old people in their childhood when exposure to nature and eating habits were more organic than nowadays.

In conclusion, the composition of the gut microbiota in the oldest-old and young groups is different, including the enterotype level. Higher species richness and diversity of the gut microbiota are characteristic of centenarians and oldest-old people with good cognitive function. Their gut microbiota contains more environmental and hereditary bacteria. Young people’s gut microbiota contains more butyrate-producing bacteria and bacteria that characterized an animal-based diet. These differences are probably due to many environment-associated factors that occurred during the development of gut microbiota in childhood as well as due to eating habits.

## Data Availability Statement

The data presented in the study are deposited in the SRA (Sequence Read Archive) repository, accession number PRJNA806961 (https://www.ncbi.nlm.nih.gov/sra/PRJNA806961).

## Ethics Statement

The studies involving human participants were reviewed and approved by the Research Ethics Committee of the University of Tartu. The patients/participants provided their written informed consent to participate in this study.

## Author Contributions

ES designed and conducted the microbiota investigation and wrote the paper. MM and RM designed the experiment and wrote the paper. ISm, TR, JŠ, and SK designed and conducted the microbiota investigation. ISo and MA conducted the interviews with the subjects. HK collected and interpreted the health data from a national database (Electronic Health Record). MV conducted the statistical data analysis. MJ conducted the bioinformatics and statistical data analysis. All authors contributed to the article and approved the submitted version.

## Funding

This study was supported by the Estonian Research Council (grant no. IUT34-19), Estonian Ministry of Education and Research (grant no. KOGU-HUMB), National Foundation of Civil Society (grant no. SMVBS17414), and Enterprise Estonia (grant no. EU48695).

## Conflict of Interest

Author MV was employed by company BioCC OÜ.

The remaining authors declare that the research was conducted in the absence of any commercial or financial relationships that could be construed as a potential conflict of interest.

## Publisher’s Note

All claims expressed in this article are solely those of the authors and do not necessarily represent those of their affiliated organizations, or those of the publisher, the editors and the reviewers. Any product that may be evaluated in this article, or claim that may be made by its manufacturer, is not guaranteed or endorsed by the publisher.

## Supplementary Material

Supplementary Table Eating habits of study subjects according to questionnaire.

Supplementary Figure Relative abundance of different butyrate producing microorganisms in oldest-old and young group (uncorrected p-values).
