# Supplementary material for: Comparative Analysis of Gut Microbiota in Centenarians and Young People: Impact of Eating Habits and Childhood Living Environment
Source: Front Cell Infect Microbiol. 2022 Mar 15;12:851404. doi: 10.3389/fcimb.2022.851404 (PMC8965453; doi:10.3389/fcimb.2022.851404)
Supplement: Supplementary Table — Eating habits of study subjects according to questionnaire. [file Table_2.docx]

| **Eating habits** | **Frequency** | **Oldest-old**  n=25 | **Young**  n=25 | **Fisher’s exact test; p-value** |
| --- | --- | --- | --- | --- |
| How often do you eat fermented food? | At least three times a day | 0 | 0 | 0.234 |
|  | Twice a day | 5 | 2 |  |
|  | Once a day | 6 | 3 |  |
|  | Less than once a day, but at least four times a week | 1 | 1 |  |
|  | Less than four times a week, but at least once a week | 9 | 13 |  |
|  | Less than once a week | 1 | 5 |  |
|  | Never | 3 | 1 |  |
|  | | | | |
| How often do you eat meat and meat products? | At least three times a day | 0 | 2 | 0.355 |
|  | Twice a day | 10 | 4 |  |
|  | Once a day | 7 | 7 |  |
|  | Less than once a day, but at least four times a week | 4 | 4 |  |
|  | Less than four times a week, but at least once a week | 3 | 5 |  |
|  | Less than once a week | 1 | 1 |  |
|  | Never | 0 | 2 |  |
|  | | | | |
| How often do you eat vegetables and salads, except  potatoes? | At least three times a day | 0 | 3 | 0.099 |
|  | Twice a day | 3 | 8 |  |
|  | Once a day | 16 | 10 |  |
|  | Less than once a day, but at least four times a week | 4 | 2 |  |
|  | Less than four times a week, but at least once a week | 2 | 1 |  |
|  | Less than once a week | 0 | 1 |  |
|  | Never | 0 | 0 |  |
|  | | | | |
| How often do you eat potatoes? | At least three times a day | 0 | 0 | **0.047** |
|  | Twice a day | 2 | 0 |  |
|  | Once a day | 9 | 4 |  |
|  | Less than once a day, but at least four times a week | 6 | 3 |  |
|  | Less than four times a week, but at least once a week | 6 | 10 |  |
|  | Less than once a week | 2 | 8 |  |
|  | Never | 0 | 0 |  |
|  | | | | |
| How often do you eat white bread, black bread, porridge? | At least three times a day | 14 | 0 | <0.001 |
|  | Twice a day | 8 | 4 |  |
|  | Once a day | 3 | 12 |  |
|  | Less than once a day, but at least four times a week | 0 | 3 |  |
|  | Less than four times a week, but at least once a week | 0 | 5 |  |
|  | Less than once a week | 0 | 1 |  |
|  | Never | 0 | 0 |  |
|  | | | | |
| How often do you eat milk and dairy products? | At least three times a day | 6 | 4 | 0.893 |
|  | Twice a day | 8 | 7 |  |
|  | Once a day | 6 | 5 |  |
|  | Less than once a day, but at least four times a week | 2 | 5 |  |
|  | Less than four times a week, but at least once a week | 2 | 2 |  |
|  | Less than once a week | 1 | 1 |  |
|  | Never | 0 | 1 |  |
