# Supplementary figures and images for: Comparative Analysis of Gut Microbiota in Centenarians and Young People: Impact of Eating Habits and Childhood Living Environment
Source: Front Cell Infect Microbiol. 2022 Mar 15;12:851404. doi: 10.3389/fcimb.2022.851404 (PMC8965453; doi:10.3389/fcimb.2022.851404)

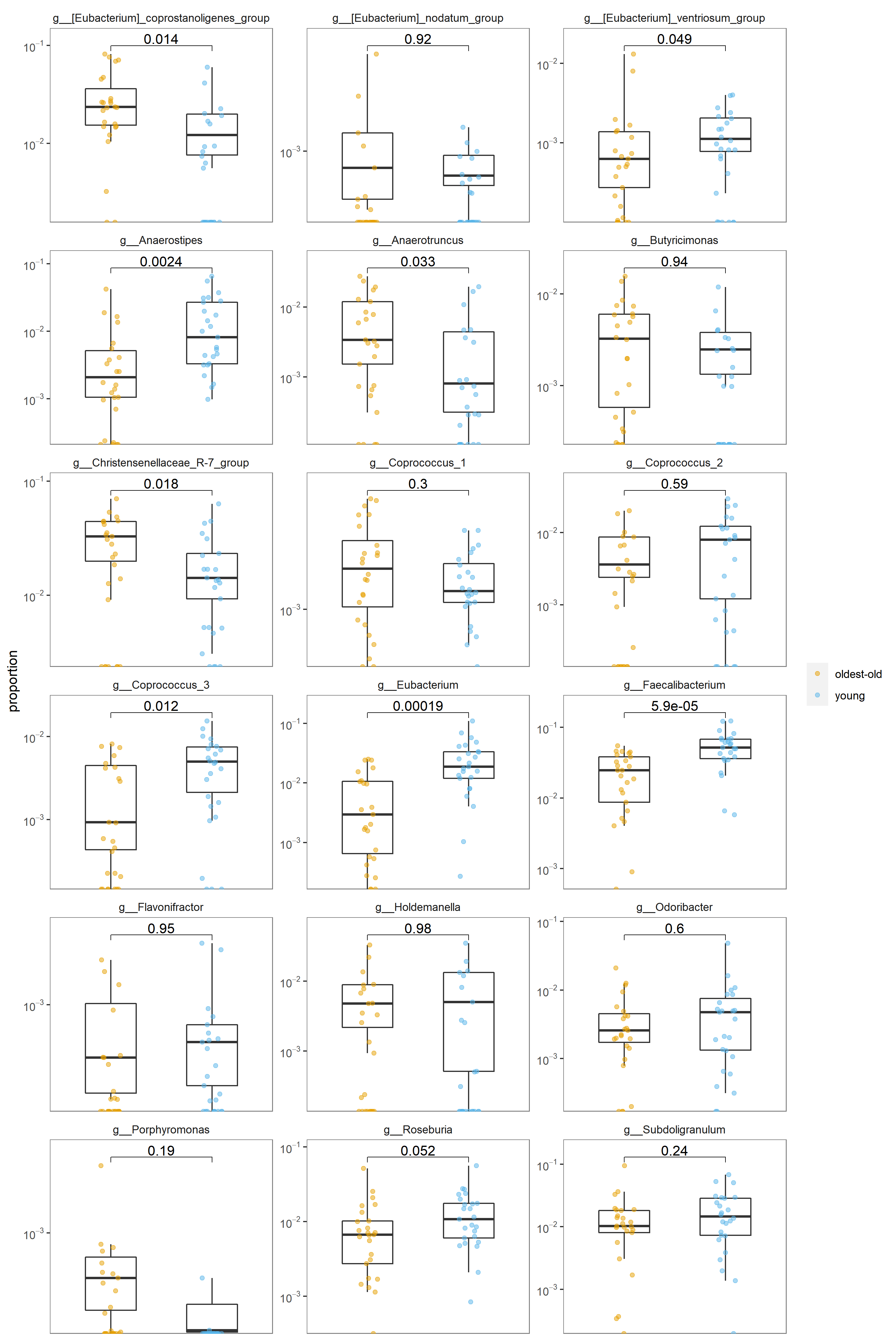

Supplement: Supplementary Figure — Relative abundance of different butyrate producing microorganisms in oldest-old and young group (uncorrected p-values). [file Table_1.docx]
